# Editorial: Multisensor Systems for Analysis of Liquids and Gases: Trends and Developments

**DOI:** 10.3389/fchem.2018.00591

**Published:** 2018-11-28

**Authors:** Larisa Lvova, Dmitry Kirsanov

**Affiliations:** ^1^Department of Chemical Sciences and Technology, University ‘Tor Vergata’, Rome, Italy; ^2^Institute of Chemistry, St. Petersburg State University, St. Petersburg, Russia

**Keywords:** chemical sensors, electronic tongue (e-tongue), chemometrics, multisensory systems, electronic nose (e-nose)

The Research Topic is devoted to the recent advances in the development of multisensor systems for analysis of liquids and gases. These artificial systems are known as “electronic tongues” and “electronic noses” since the inspiration for their development was found in human taste and olfaction systems, and they have been actively employed in the last two decades as effective, simple, fast, and inexpensive analytical tools for various analytical tasks. The popularity of these tools is mainly due to the two following reasons: (1) they allow quantification of complex integral quality parameters in liquids and gases, like e.g., taste descriptors; (2) they are typically much more simple and less expensive compared to the majority of modern instrumental analytical methods, yet capable of providing meaningful and important information on quantitative and qualitative composition of the samples.

Despite the high interest and wide distribution of artificial sensory systems nowadays, we cannot say that the field is fully mature: there are still many aspects to be developed and understood, from sensing materials composition, system construction and employed experimental set-ups to the details on measuring techniques, data processing methods, calibration procedures, and practical applications. The dedicated efforts are necessary for the understanding of all these aspects in order to put the multisensor systems into a common routine laboratory practice. Of course, the present research collection does not cover the whole range of advancements in the field, however it highlights certain very interesting contributions on diverse aspects of electronic tongues and noses and we hope, this will inspire further interest and new research efforts in this exciting area.

The Research Topic contains the collection of 14 original contributions: 11 research papers, two reviews, and one mini review dedicated to the different aspects related to the development and application of chemical sensors in the multisensor analysis. The mini review of Cuypers and Lieberzeit reports on the sensor arrays combining chemoselectivity and chemometrics based on biomimetic approaches in order to maximize chemical selectivity of a final device. The main aims of such systems development are: the improvement of analytical quality of quantitative data and a deeper understanding of the natural olfaction processes. The review of Rudnitskaya describes various methods for drift correction and calibration update in multisensor systems—a very important issue both for routine practical application of such systems and for scientific experiments. The contribution from the group of Sorvin et al. represents an overview of solid-state polyaniline-based potentiometric sensors and multisensor systems doped with thiacalixarene receptors for analysis of beverages. The application of these compounds has a great potential in multisensor analysis. In the study of Mascini et al. group ZnO nanoparticles were modified with computationally selected peptides, which is quite a new concept in sensor studies gaining popularity. Such a modification allowed constructing the gas sensors with pronounced sensitivity toward volatile organic compounds. The group of Garcia-Hernandez et al. which has done a lot of research in voltammetric multisensor systems for wine analysis here reports on an interesting development of this concept for monitoring the phenolic ripening of red grapes. Quite a timely research from the group of Gaál et al. describes the way we can benefit from 3D printing technology in multisensor system construction. Drozd et al. together with colleagues reports on surface plasmon resonance biosensing platforms that can be adopted in the future for multisensing in biological applications. The contribution from Fernandez et al. addresses the field where much work remains to be done—the evaluation of the analytical figures of merit for multisensor systems. The paper deals with the protocol to assess the resolving power of sensor array. The studies in this direction are absolutely necessary for the development of the meaningful comparison protocols to select between various multisensor systems. The contribution of Lvova et al. is devoted to the application of heteroatomic macrocyclic fluorophores (diaza-crown ether, metallocorrole and pyridinophans) for development of a fluorescent sensor array with a nice potential for environmental applications. The paper by Nikolaev et al. reports on a new procedure to develop chip-based nanoelectrochemical sensor arrays based on the directed electrochemical nanowire assembly (DENA) and their utility for non-enzymatic analysis of hydrogen peroxide, glucose, and ethanol. Such systems can be especially attractive for healthcare applications. The group of Röhlen et al. has developed the concept of hybrid biosensor with application for biogas process monitoring. Normally biosensors are not in the focus of multisensor systems development as they typically have quite high selectivity; however the benefits of multisensor arrangement can become important in complex multicomponent media. Pennazza et al. has shown how the advanced electronics and additional operative conditions can improve the analytical performance of sensor arrays in particular based on the advances in the electronics for cyclic voltammetry. The contribution from the group of Tu et al. describes the development of quite popular concept of aptamer-based sensing toward the detection of hazardous mercury ions in environmental samples. Avossa et al. has investigated the idea of tuning working temperature to modify both the sensitivity and the selectivity of nanocomposite conductive gas sensors showing a good promise for practical application of this principle in air pollutant detection.

The Editors hope that this collection will be of a great interest to the Frontiers audience and will inspire significant progress in the field of multisensor systems for chemical analysis.

## Author contributions

All authors listed have made a substantial, direct and intellectual contribution to the work, and approved it for publication.

### Conflict of interest statement

The authors declare that the research was conducted in the absence of any commercial or financial relationships that could be construed as a potential conflict of interest.

